# Leadership in global health: the case of Ciro de Quadros, a testament to values, valor, and vision

**DOI:** 10.26633/RPSP.2017.149

**Published:** 2017-12-20

**Authors:** Jon Kim Andrus

**Affiliations:** 1 Division of Vaccines and Immunization, Center for Global Health Division of Vaccines and Immunization, Center for Global Health, University of Colorado, Denver DenverColorado United States Adjoint Professor, Senior Investigator, and Director, Division of Vaccines and Immunization, Center for Global Health, University of Colorado, Denver, Colorado, United States; Former Director and Senior Technical Advisor of Extended Program of Immunization and Deputy Director, Pan American Health Organization, Washington, DC, United States.

When Dr. Bill Foege wrote “When Words Fail,” he was referring to how difficult it was for him to describe adequately, in written words, all the effort that is involved from the scientific conceptualization of a new vaccine, to eventual bench discovery and development, to the training and supply chain logistics, and ultimately to the moment the needle pricks the skin to save a life of a child (1). He called it the “chain of perfection”. He was “at a loss” to describe this cascade of events with due justice. I have been asked to describe the leadership attributes of Dr. Ciro de Quadros as a case study in best practice and lessons to be learned. Similarly, I too am at a loss. Simply put, Ciro broke the mold.

I should disclose that what I am about to write is influenced by decades of either working directly for Ciro, as was the case during the polio eradication era in the Americas, to collaborating with Ciro more recently on various projects that included measles and rubella elimination, the introduction of new vaccines, and surveillance of infectious diseases. The work evolved into a relationship that bridged from professional mentorship, to actual friends with a deep sense of admiration, love, and mutual respect.

In global health, leadership is somewhat like the “self-actualization” of public health practice. The “leader” must have all those attributes that embodies a fully realized individual, positioned in the system to make things happen, such as in Ciro’s case, to help create a world of equitable access to life-saving vaccines. Ciro always kept such a vision in mind. He was unrelenting when seemingly unsurmountable forces impeded his way. Like a hurricane, he would uproot naysayers, but with a clear sense of knowing exactly where he was going. I will attempt to describe what made Ciro this “force of nature”, including, perhaps, what arguably may have been some of his deficiencies. The three “V’s”, values, vision, and valor, have been used to describe broadly the attributes of effective leaders. For Ciro, volumes could be written on each, especially if we think of his career in its entirety, spanning more than four decades.

## VALUES

As a boy growing up on his father’s farm in Porto Alegre, Brazil, Ciro learned very early about the value of hard work. The family’s labor under his father’s guidance contributed directly to survival of the family business during difficult times. His calling, though, was not the manual labor and physical toll of cattle ranching. His calling and vocation would eventually be medicine, public health, and prevention. Initially, he trained to be a psychiatrist. Smallpox eradication and public health would come later. Regardless of his intent, hard work was a key guiding principle.

Later, he demanded the same work ethic of each of his team members whom he managed over the years. He was firm to the point that to some he was like a slave driver. Such firmness was reinforced by his own example. He never demanded what he would not do himself. A former Peace Corps volunteer who worked for Ciro in Ethiopia during smallpox described to me once how Ciro sent him out on a donkey alone to evaluate how work was progressing in a rather isolated village. After a night camping under the stars alone, the volunteer decided to sleep a bit more after the early sunrise. While resting under his blanket, the volunteer was surprised by the approach of Ciro catching up to him during his few precious moments of additional rest. Clearly, the volunteer was impressed with Ciro’s fortitude. Thereafter, he vowed not to let Ciro down. They became dynamic colleagues.

His study of human nature through psychiatry benefited his interpersonal skills. He often showed affection with a big hug for hard work. But, Ciro was decisive, arguably to some almost to a fault. There was no in-between, no compromise on certain key issues, such as the polio eradication vaccination strategies. Some would often complain that he was too dogmatic. To be effective, the Peace Corps volunteer in Ethiopia recognized that certain standards needed to be maintained. One untold benefit was the acceptance of the local citizens. Ciro was an “outsider”, but he complied with, and adjusted to, the demands of the local circumstances. Sitting in Addis Ababa would not eradicate smallpox in Ethiopia. Getting out to the remotest villages, where the most difficult challenges occurred, leading by example, was Ciro’s style. The Ethiopians took note. They would forever be his friends and colleagues.

Certainly, among his many leadership traits was his ability to persevere, not just over years, but over decades. He was the ideal eradicator. To get to the target, he would never take no for an answer. He was a stout polio champion who always believed the world could do it, despite all the setbacks. With each setback he had the answers, not always appreciated by all. But, he persevered, he never gave up. Except in the post-eradication world, in his mind the killed, injected polio vaccine never had a place in stopping transmission of wild poliovirus.

Ciro also had a deep sense of fairness when it came to social justice. Very early on he was among the top leaders in global health to champion the cause of women in leadership positions. He may have even been the first to hire a woman as the Director of a WHO Regional Expanded Program of Immunization (EPI). Many of the young field staff that he trained and groomed through the ranks were among the most talented women in Latin America and the Caribbean. They were all hard-working and dedicated to the cause of public health. Historically, during a time when PAHO was predominately male dominated, Ciro had no equal in breaking down the barriers of gender bias.

Ciro prided himself on never being late, always keeping to the agreed upon schedule. Many programs today would benefit from this sense of urgency. Like many leaders, Ciro was inclined to delegate the most urgent tasks to the “work horses” on the team, to those he knew would deliver the tasks in the time he needed them. The 1990 target for polio eradication in the Americas was not to be missed at any cost. The fact that the initiative was 8 months, 23 days late in reaching the eradication goal, was in a way disappointing to Ciro. Even when over-tasking, however, he would never heap work on anyone that he would not personally do himself.

## VALOR

PAHO has a film clip in its archives of Ciro in Ethiopia during his smallpox days. There is no voice-over. He is riding a donkey into a village to investigate contacts of a smallpox case in a neighboring village. The clip goes on to show Ciro interviewing what appear to be the village leaders. As Ciro described it to me, the leaders point to the direction where they think a potential contact of the smallpox case may have traveled. They were also looking at a map. Clearly, the contact had left the village and no one knew his exact whereabouts. Ciro was on a mission to be sure that no contacts were lost. He had no other choice but to continue his trek, not knowing where he would ultimately end. If infected and contagious, a missed contact could set the global smallpox program back months.

One day in my office at the Pan American Health Organization (PAHO), DA Henderson and Ciro watched this same film clip. DA was interviewing Ciro at the time as part of his effort to document the history of smallpox eradication. The two reminisced about old times. As the global director of the smallpox eradication program, DA was Ciro’s boss. During the conversation, Ciro downplayed the concern about personal safety and the absolute uncertainty of not knowing where he was going to sleep that night. The conversation drifted to the civil war that had been taking place at the time in Ethiopia. DA repeatedly tried to get Ciro to describe the fears he may have had. Ciro dodged such questions. He did say that he was not particularly enthusiastic about the frequent helicopter rides he had to take to get around the country. The helicopter was particularly exposed to unfriendly fire. Ciro explained how he would be able to go home after the last smallpox case, his Ethiopian co-workers would have to remain, continually threatened by local circumstances. They had no escape hatch button, no home across the ocean, free from conflict. For that he admired them, feeling that they were certainly more deserving of accolades than him.

Perhaps more remarkable, was Ciro’s courage to put his own reputation on the line for equity and health. He was a champion of the underserved, regardless of race, religion, country of origin, or socio-economic status of the individual. In fact, I knew of no one on his teams over the years who had dis-similar views, but no one put their name or their reputation on the line, as only Ciro could. Some have referred to this type of courage as, “falling on your sword.” Indeed, such actions were not always popular. Mahatma Gandhi once said, “I am hard hearted enough to let the sick die… if you can tell me how to prevent others from getting sick.” Ciro had that kind of hardness with a greater picture of a healthier world in mind.

In 1989, when I first arrived to PAHO to work for Ciro on polio eradication, my office was next to his. On one of my first days, I distinctly remember Ciro yelling on his phone next door. He could be heard far down the hallway, “I will never take you to a country again. That is not how you treat a Minister!!! Who do you think you are? That is not how you treat people, especially a Minister of Health!!!” After slamming down the phone, Ciro marched out of his office and held court in the hallway to any who would listen. “Who does Albert think he his?” Of course, he was referring to Albert Sabin, the discoverer of the oral polio vaccine, the vaccine that would be used to eradicate polio in the Americas, and eventually in many parts of the world. Albert’s stature in the United States was extraordinary, he went on to be buried in Arlington Cemetery, an incredible honor. Of course, Ciro was unfazed by his stature.

I cannot count the occasions over the years where I witnessed Ciro being equally blunt to other global public health leaders, especially those who were from affluent countries with little to no field experience working in developing countries. As an example, he would not hesitate to say to a global agency head, face-to-face, that they were too aligned with industry. His point being that some leaders were more inclined to protect the profits of industry over what it would take to reach every impoverished child on the planet. Clearly, he knew industry needed to regain the return on their investment, but not disproportionately, especially at the disproportionate cost of losing the opportunity to save more lives.

Some producers would do what he perceived to be unacceptable practices. For example, he created and managed the PAHO Revolving Fund (RF). The RF used pooled vaccine demand and forces of economies of scale to negotiate prices from the producers. On behalf of PAHO countries the RF purchased vaccines based on the negotiated prices. Some producers tried to dissuade countries from using the RF. In so doing, the RF would lose its economies of scale purchasing power. He heroically battled many vaccine producers over the years not to undermine the RF, but to remain committed to its enormous potential as a public health tool. He adeptly forged relationships among the producers that were critical for the success of the program, especially for the provision of a safe, affordable vaccine supply. A prime example was the role of the Serum Institute of India. The Institute provided measles-rubella antigen containing vaccine at an affordable price that would later help countries of the Americas eliminate measles and rubella.

## VISION

Ciro was the consummate visionary. He knew what needed to be done, and equally important how to get there. He was never one for merely stating that a problem existed. He always offered a solution. Nor did he ever tear down a county’s program only to gain more spotlight for what he had to say. Indeed, he had the destination and the roadmap on how to get there. For the values described above, the engine that propelled his efforts was vaccine. Arguably, Ciro knew more than anyone how priceless vaccines were for saving lives. One could not talk abouthealth for all without Ciro talking about the role of vaccines and immunization, the same for primary care, outbreak response, emerging infections. Ciro had each of these themes on his roadmap.

Ciro understood that every country needed to have an annual national plan of action that was regularly monitored and reviewed, always grounded in accountability. He made sure that every country reported annually on their plans of action. He praised the good work while diplomatically acknowledging any shortcomings. He was instrumental in helping create the policy that provided the underpinnings for every national plan. He did this by creating the PAHO Technical Advisory Group (TAG) for Vaccine Preventable Diseases. Although several prominent American epidemiologists served on PAHO’s TAG, Ciro often fought against any reflex that just because a certain policy was enacted in the United States, that same policy should automatically be applied to countries of Latin America and the Caribbean.

Whole cell pertussis vaccine, discontinued in the United States, is still the backbone of pertussis prevention and control in PAHO. As a result, PAHO has been able to avoid the pertussis outbreaks that have recently plagued the United States because of its policy shift from whole cell to a subunit vaccine with fewer side effects of soreness at the injection site, but unfortunately with less duration of protection. Ciro saw that soreness of the arm was nothing if the vaccine could save more lives. Of course, the circumstances were very different. The culture of litigation and anti-vaccine group movement are not nearly as prominent in countries of Latin America and the Caribbean as they are in the United States.

From the very beginning he also galvanized buy-in from all the departments within PAHO. He wanted EPI to be PAHO’s program, not one of any one country, not one of any one man, including Ciro. He is credited for being the “grandfather” of EPI in the Americas. He was a genius to early on recognize that implementing a technical plan, coupled with an assured supply of affordable vaccines, was a game changer. As mentioned, he created PAHO’s RF for purchase of vaccines and supplies, using pooled demand to create economies of scale within the framework of competitive markets. The result was an affordable price for regardless of who you were, or were you lived. Arguably, no other purchasing mechanism has come close to the impact created by the more 45 years of service the RF has had in the Americas. I once asked him before he died what he was most proud of with regards to his life’s work, he did not hesitate to say the Revolving Fund.

Vaccine prices were only part of the problem. Ciro knew that to sustain progress, countries had to own their national programs. He understood the whims and conditionalities of external donors, and knew early on that reliance of such donors would potentially undermine, if not destroy, national immunization programs. He feared national immunization programs were at risk to being manipulated or “owned” by outsiders.

To that end, Ciro championed the development of vaccine legislation, country by country. He had his field staff actively engaged and reporting to him regularly on progress to vaccine legislation development. A vaccine law that required a country to have a line item for vaccine purchase in their annual budgets, to have a national plan of action reviewed annually, and to have an effective leader running the program, were key components of ideal legislation. The data today support this approach. Countries overwhelmingly purchase their vaccines from national budgets with little to no reliance on external funds. Haiti, the poorest country in the Region, has also made progress in this area. There are no words to describe what began as an idea in Ciro’s mind, some 45 years ago, to what we see today in terms of country ownership. Ciro was a force of nature with a clear sense of direction regarding country ownership and a road map of vaccine laws on how to get there.

In the final years of his life, Ciro spent most of his time helping steward in the Decade of Vaccines. The WHO Global Vaccine Action Plan (GVAP) for the Decade of Vaccines is really a testament to Ciro de Quadros. Those elements that Ciro championed throughout his life-time, are all front and center in GVAP, including country ownership; new vaccine introductions; polio, measles, and rubella eradication; surveillance of vaccine preventable diseases; and program accountability and leadership, to name a few.

In mid-2013, Ciro was diagnosed with an incurable cancer. Approximately two months before he died PAHO paid special tribute to Ciro with a life-time achievement award. He was extremely appreciative to receive such recognition from his friends and colleagues while he was still alive.

In summary, Dr. Ciro de Quadros ranks among the top leaders in the history of public health. He had a positive health impact on millions of lives of people around the world, particularly those most impoverished and underserved. His leadership style embodied how to get things done, especially when all odds were against him. To that end, many governments have recognized him: Thailand with its Prince Mahidol Award, Asia’s most prestigious award in public health; Spain with its highest award in public health; Mexico with membership to its National Academy of Medicine; and Chile with its highest national award, to name a few. His leadership journey was life-long. John Maxwell describes such a journey quite succinctly, “learn, earn, and return.” Ciro’s early years spent learning in Brazil and Ethiopia provided a foundation over his professional career of field experience that enabled him to deliver in practice with unprecedented credibility. He earned that credibility working in very challenging field situations. With each phase of his leadership development he returned to others by teaching them to lead. The footprint Ciro left for better health on the planet is immeasurable. Words cannot adequately describe all the benefits resulting from his life’s work. Indeed, what can be said about a man, who suffering from the ravages of cancer, held his last staff meeting from his bed, five days before he died? The “bottom line” was always ahead of him, more work to be done, more lives to save.
